# Heparanase Is a Putative Mediator of Endothelial Glycocalyx Damage in COVID-19 – A Proof-of-Concept Study

**DOI:** 10.3389/fimmu.2022.916512

**Published:** 2022-06-10

**Authors:** Carolin Christina Drost, Alexandros Rovas, Irina Osiaevi, Matthias Rauen, Johan van der Vlag, Baranca Buijsers, Rustem Salmenov, Alexander Lukasz, Hermann Pavenstädt, Wolfgang A. Linke, Philipp Kümpers

**Affiliations:** ^1^ Department of Medicine D, Division of General Internal and Emergency Medicine, Nephrology, and Rheumatology, University Hospital Münster, Münster, Germany; ^2^ Department of Medicine A, Division of Hematology, Oncology, Hemostaseology and Pneumology, University Hospital Münster, Münster, Germany; ^3^ Department of Nephrology, Radboud Institute for Molecular Life Sciences, Radboud University Medical Center, Nijmegen, Netherlands; ^4^ Institute of Physiology II, University of Münster, Münster, Germany

**Keywords:** COVID-19, heparin, heparanase (HPSE), videomicroscopy, endothelial glycocalyx (EG)

## Abstract

Coronavirus disease 2019 (COVID-19) is a systemic disease associated with injury (thinning) of the endothelial glycocalyx (eGC), a protective layer on the vascular endothelium. The aim of this translational study was to investigate the role of the eGC-degrading enzyme heparanase (HPSE), which is known to play a central role in the destruction of the eGC in bacterial sepsis. Excess activity of HPSE in plasma from COVID-19 patients correlated with several markers of eGC damage and perfused boundary region (PBR, an inverse estimate of glycocalyx dimensions of vessels with a diameter 4-25 µm). In a series of translational experiments, we demonstrate that the changes in eGC thickness of cultured cells exposed to COVID-19 serum correlated closely with HPSE activity in concordant plasma samples (R = 0.82, P = 0.003). Inhibition of HPSE by a nonanticoagulant heparin fragment prevented eGC injury in response to COVID-19 serum, as shown by atomic force microscopy and immunofluorescence imaging. Our results suggest that the protective effect of heparin in COVID-19 may be due to an eGC-protective off-target effect.

## Introduction

Coronavirus disease 2019 (COVID-19) caused by severe acute respiratory syndrome coronavirus 2 (SARS-CoV-2) is presenting as a systemic disease associated with vascular inflammation and endothelial injury (1–3). Using novel, quantitative sublingual video microscopy, we were able to show that severe damage (thinning) of the endothelial glycocalyx (eGC) predicted 60-day in-hospital mortality in our cohort of COVID-19 patients (4).

The endothelial glycocalyx (eGC) is a delicate gel-like layer coating the luminal surface of the vascular endothelium (5, 6). It is up to 3 µm thick, largely consists of highly sulfated glycosaminoglycans and proteoglycans, and it plays a pivotal role in the maintenance of microcirculatory homeostasis (7, 8). Specifically, the eGC acts as a negatively charged “firewall” to reduce leukocyte-endothelial-interactions (9). Its carbohydrate-rich matrix provides resistance to water permeability and contributes to the proportion of albumin molecules “reflected” back into plasma by the vessel wall (10, 11). Beyond that, the glycocalyx contributes to the regulation of the redox state and is crucially involved in the mediation of shear-induced nitric oxide release as well as physiologic anticoagulation (8, 12, 13).

At least in bacterial sepsis, the final common pathway of eGC damage appears to be remarkably consistent: the activation and release of the heparan sulfate (HS)-degrading enzyme heparanase (HPSE) (14, 15). Besides cleaving HS from the cell surface, HPSE also enhances shedding of transmembrane HS proteoglycan syndecan-1 (Syn-1) by upregulating the expression of matrix metalloproteinase 9 (MMP9), which is a syndecan sheddase. Therefore, HPSE (in)directly contributes to increased HS and Syn-1 plasma levels, which are both markers of endothelial glycocalyx degradation. The aim of this translational proof-of-concept study was to investigate the role of HPSE in COVID-19-induced eGC damage.

## Methods

### Study Design and Study Population

Sublingual video microscopy and blood sampling were performed in 16 adult PCR-confirmed COVID-19 patients, prospectively enrolled from May to June 2020 at the intensive care units (ICU) of the University Hospital Münster and three local teaching hospitals in a non-consecutive manner. Plasma samples were obtained, centrifuged, and stored at -80°C until analysis. Twelve apparently healthy, age-matched volunteers served as controls (**Table 1**). Some of the participants were already included in a previous study (4). The study was performed in accordance with the Declaration of Helsinki and approved by the Ethics Committee of the General Medical Council Westfalen-Lippe and the WWU Münster, Germany (file number: amendment to 2016-073-f-S). Written informed consent to participate has been obtained from all individuals.

**Table 1:** Baseline characteristics.

Variable	Healthy Controls [*in vitro* subgroup]	COVID-19 ICU [*in vitro* subgroup]	*P* value* [subgroup]
Number of participants (n)	12 [5]	16 [6]	- [-]
Female sex (n; %)	6 (50) [3 (60)]	1 (6.3) [1 (17)]	0.008 [0.14]
Age (years, median (IQR))	53 (45-60) [57 (53-70)]	62 (56-72) [59 (55-67)]	0.06 [0.89]
BMI (kg/m^2^, median (IQR))	25 (23-29) [25 (24-33)]	27 (24-32) [28 (27-33)]	0.39 [0.43]
SOFA score (pts, median (IQR))	- -	9.5 (5.3-15.8) [9.5 (4.5-15.75)]	- [-]
LWMH/Heparin (n; %)	- -	11 (68.75) [4 (66)]	- [-]
Invasive mechanical ventilation (n; %)	- -	14 (87.5) [6 (100)]	- [-]
In-hospital mortality (n; %)	- -	6 (37.5) [4 (67)]	- [-]
**Sublingual microscopy** (median (IQR))			
PBR_4-25_ (µm)	2.18 (2.1-2.23) [2.11 (2.06-2.21)]	2.39 (2.13-2.52) [2.44 (2.25-2.58)]	0.016 [0.028]
**Endothelial glycocalyx markers** (median (IQR))			
Heparanase activity (AU)	0.92 (0.68-1.23) [0.99 (0.78-1.72)]	2.26 (1.67-2.97) [2.58 (2.05-3.77)]	<0.0001 [0.015]
Heparan sulfate (AU)	11.6 (2.2-80.0) [28.4 (11.01-157.6)]	154.5 (85.3-408.8) [127.4 (62.2-258.5)]	0.0004 [0.33]
Syndecan-1 (ng/ml)	18.2 (15.1-24.91) [19.01 (15.38-52.9)]	219.8 (161.2-249.7) [248.9 (244.5-256.2)]	<0.0001 [0.004]
Hyaluronic acid (ng/ml)	78.74 (75.48-85.63) [75.95 (73.08-81.91)]	240.6 (173.7-541.9) [213.5 (165.6-346.0)]	<0.0001 [0.004]
**Laboratory values **(median (IQR))			
CRP (mg/dl)	<0.5 [<0.5]	14.2 (11.0-22.3) [11.1 (6.6-17.6)]	<0.0001 [0.008]
IL-6 (pg/ml)	<2 [<2]	79.5 (52.3-200.8) [80 (49.0-107.0)]	<0.0001 [0.004]
PCT (ng/ml)	<0.5 [<0.5]	1.27 (0.47-5.05) [1.7 (0.17-8.81)]	0.031 [0.68]
Angpt-2 (ng/ml)	1.04 (0.54-2.07) [1.74 (0.74-2.13)]	6.44 (4.48-6.62) [6.44 (4.83-6.44)]	<0.0001 [0.004]
aPTT (s)	36 (34.5-37) [37 (34-37.5)]	42.5 (39.25-56.5) [46.5 (40.5-61.5)]	<0.0001 [0.004]
D-Dimers (mg/l)	<0.5 [<0.5]	4.02 (2.44-8.64) [6.7 (2.51-11.71)]	<0.0001 [0.069]

Differences between groups were calculated by Mann-Whitney U test or Chi-square test, as appropriate.

^*^ p value between healthy controls and COVID-19 ICU.

COVID-19, Coronavirus disease 2019; BMI, Body mass index; SOFA score, Sequential Organ Failure Assessment score; LMWH, low molecular weight heparin; IQR, interquartile range; PBR, Perfused boundary region; MVHS, Microvascular Health Score; AU, arbitrary unit; CRP, C-reactive protein; IL-6, Interleukin-6; PCT, Procalcitonin; Angpt-2, Angiopoietin-2; aPTT, activated partial thromboplastin time.

### *In Vivo* Assessment of Sublingual Glycocalyx Dimensions

Real-time intravital microscopy was performed at the bedside with a sidestream dark field (SDF) camera (CapiScope HVCS, KK Technology, Honiton, UK) to visualize the sublingual microvasculature (microvessel diameter 4–25 µm) as reported previously in detail (16, 17). In brief, the SDF camera uses green light emitting stroboscopic diodes (540nm) to detect the hemoglobin of passing red blood cells (RBCs). Image acquisition and analysis was performed by GlycoCheck™ Software (Microvascular Health Solutions Inc., Salt Lake City, UT, USA). It detects the dynamic lateral RBC movement into the glycocalyx, which is expressed as the perfused boundary region (PBR, in µm). An altered or degraded glycocalyx allows more RBCs to penetrate deeply toward the endothelial surface, with a consequent increase in the PBR. In every patient, we performed two complete measurements which were averaged to account for spatial heterogeneity of the sublingual microvasculature.

### HPSE Activity and HS Competition Assay

The activity of HPSE in EDTA plasma was determined by a heparanase activity assay (Amsbio, Abingdon, UK, Cat# Ra001-02-K) according to the manufacturer’s instructions but using an in-house developed HPSE buffer (18). The dose dependent inhibition of HPSE with HS isolated from bovine kidney was assessed with an in-house developed HPSE activity assay [11]. HS in EDTA plasma samples was quantified by a previously described HS competition assay. Importantly, this assay is specific to HS, therefore the measurement is not affected by the presence of heparin (18).

### Quantification of eGC Components and Angiopoietin-2

Plasma levels of Syndecan-1 (Syn-1; Diaclone, Besançon, France, Cat# 950.640.096), hyaluronic acid (HA; Echelon Biosciences Inc., Salt Lake City, UT, USA, Cat# K-1200-) and Angiopoietin-2 (Angpt-2; R&D Systems, Oxford, United Kingdom, Cat# DANG20) were measured using commercially available enzyme-linked immunosorbent assay (ELISA) kits as described previously (17, 19).

### Atomic Force Microscopy and Confocal Immunofluorescence Microscopy

Atomic force microscopy using live-cell nano-indentation technique (AFM; Nanoscope V Multimode AFM, Veeco, Mannheim, Germany) and confocal immunofluorescence microscopy (Leica DMI 6000B-CS/TCS SP8 laser confocal microscope, Leica, Wetzlar, Germany) of HS staining were performed on the human umbilical vein endothelial cell line EA.hy926 essentially as described (Amsbio, Ab Heparan sulfate, Cat# 370255-1, RRID : AB_10891554; Jackson ImmunoResearch Labs, Alexa fluor 488 goat anti-mouse IgG antibody, Cat# 115-545-146, RRID : AB_2307324) (20, 21). Cells were grown in DMEM (Gibco™; Cat# 52100047) supplemented with 10% fetal bovine serum (SigmaAldrich; Cat# S0615-100ML) and 1% penicillin/streptomycin (Biochrom; Cat# A2212) at 37°C in a 5% CO_2_ enriched environment for at least 3 days until reaching confluence. Intensity analysis was performed with ImageJ software (version 1.51p 22, NIH) as previously described (20, 21). The non-anticoagulant N-desulfated re-N-acetylated heparin (NAH), a very potent and well characterized heparanase inhibitor (12), was obtained from Iduron (Cheshire, United Kingdom, Cat# DSH004/Nac).

### Statistical Analysis

Values are presented as absolute values with median and inter-quartile range (IQR). The non-parametric Mann-Whitney U test and the Chi-Square test were used to compare parameters between groups. *In vitro* data are presented as means and standard error of the mean (SEM) unless otherwise stated. Correlation between PBR and eGC thickness was assessed by Spearman correlation coefficient. To account for both the number of observations from a single experiment and the number of experiments, differences in glycocalyx thickness were tested with a nested analysis of variance and Tukey’s *post-hoc* test. All tests used were two sided, and statistical significance was set at p<0.05. SPSS (IBM Corporation, Armonk, NY, USA, v.26, RRID : SCR_016479) and GraphPad Prism (GraphPad Prism Software Inc., San Diego, CA, USA, v.8.4.3, RRID : SCR_002798) were used for statistical analyses.

## Results

**Figure 1:**
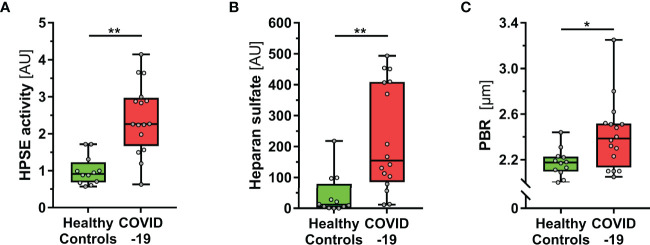
COVID-19 patients show elevated HPSE activity, and damaged eGC *in vivo*. **(A-C)** Boxplots showing **(A)** heparanase (HPSE) activity, **(B)** heparan sulfate (HS) and **(C)** perfused boundary region (PBR; an inverse estimate of the sublingual endothelial glycocalyx thickness) in healthy subjects (n = 12) and COVID-19 patients at the ICU (n = 16). Differences between groups were calculated by Mann-Whitney U test. *p < 0.05; **p < 0.001.

COVID-19 patients at the ICU had a median (IQR) Sequential Organ Failure Assessment (SOFA) score of 9 (5 – 15), were predominantly intubated (14/16, 88%) and showed an in-hospital mortality of 38% (6/16). Compared to healthy subjects, COVID-19 patients showed a significantly higher HPSE activity in plasma (**Figure 1A**). Accordingly, increased levels of HS (the main substrate of HPSE), HA and Syn-1 demonstrated eGC shedding in COVID-19 (**Figure 1B**, **Table 1**). This finding was further confirmed by high PBR values (i.e., a thin eGC) measured in COVID-19 patients (**Figure 1C** and **Table 1**). All markers of eGC damage correlated with disease severity, as measured by SOFA score (HPSE: *R*=0.6, *P*<0.001; HS: *R*=0.66, *P*<0.001; HA: *R*=0.8, *P*<0.001; Syn-1: *R*=0.81, *P*<0.001; PBR: *R*=0.38, *P*<0.05).

**Figure 2:**
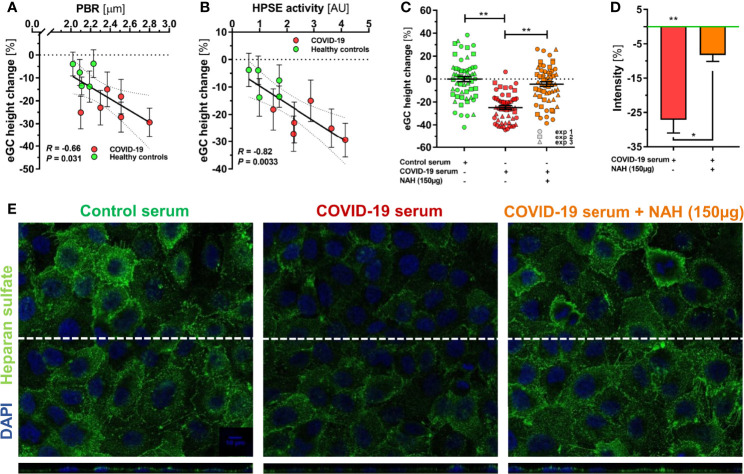
HPSE is a putative mediator of eGC damage in COVID-19. **(A-C)** Sera from a randomly selected subgroup of 5 healthy controls and 6 COVID-19 patients were sterile-filtered and incubated (5%) on the human umbilical vein endothelial cell line EA.hy926 for 60 min. Endothelial glycocalyx (eGC) thickness was assessed by atomic force microscopy (AFM) using a dedicated nano-indentation protocol. Scatter dot plot showing the association between AFM-derived eGC (*in vitro*) decline and corresponding **(A)** PBR-values (*in vivo*) and **(B)** HPSE activity for the individuals from the subgroup. Each dot represents the mean ± SEM (standard error of mean) of two independent AFM experiments (consisting of ≥ 4 indentation curves in each of ≥ 8 different cells) for each individual serum. Incubation without human serum served as control. Correlation was assessed by Spearman correlation coefficient. **(C)** Dot plots from three independent AFM experiments (pooled serum from subgroups) showing values with mean ± SEM. Each dot represents the mean of ≥ 4 indentation curves per cell. Heparanase was blocked by N-desulfated re-N-acetylated heparin (NAH; 150 µg). Differences between groups were calculated with nested ANOVA and Tukey’s *post-hoc* test. Intensity analysis of heparan sulfate-stained EA.hy926 cells **(D)** and representative immunofluorescence images **(E)** after treatment with 5% control serum or COVID-19 serum ± NAH (150 µg) for 60 min. Values are normalized to control serum (zero line) and differences between groups were assessed with nested ANOVA and Tukey’s *post-hoc* test. Data are presented as mean ± SEM. *p < 0.05; **p < 0.001.

To further validate our findings, we exposed endothelial cells (ECs) to randomly selected sterile-filtered sera (5%; diluted in buffer) from 6 COVID-19 patients and 5 healthy subjects for 60 minutes. COVID-19 serum, but not serum from healthy subjects, caused a distinctive decrease of eGC thickness *in vitro* [-7 (4-14) % *vs*. -24 (17-28) %, *P* < 0.01]. The delta changes of eGC thickness measured by AFM *in vitro* strongly correlated with concordant PBR values (*R* = -0.66, *P* = 0.03) obtained by videomicroscopy *in vivo* (**Figure 2A**), indicating that the eGC damage seen in COVID-19 can be quantitatively reproduced in cultured ECs.

Delta changes of eGC thickness measured by AFM also correlated closely with the respective plasma HPSE activity in the samples (*R* = 0.82, *P* = 0.003) (**Figure 2B**). Competitive inhibition of heparanase by the nonanticoagulant heparin fragment NAH completely prevented the decline in eGC thickness in response to COVID-19 serum (**Figure 2C**). Similarly, immunofluorescence imaging of the eGC showed a marked decrease in HS positivity and surface coverage in ECs incubated with COVID-19 serum, whereas COVID-19 serum supplemented with NAH caused only a very slight loss in staining intensity and coverage compared to incubation with serum from healthy controls (**Figures 2D, E**).

In the total cohort, HPSE correlated well with inflammatory mediators, such as C-reactive protein (*R* = 0.78, *P* < 0.001), interleukin (IL)-6 (*R* = 0.63, *P* < 0.001), and Angpt-2 (*R* = 0.61, *P* < 0.001) as well as the eGC marker Syn-1 (*R* = 0.71, *P* < 0.001). Surprisingly, however, HPSE activity did not correlate with the PBR (*R* = 0.11, *P* = 0.58) or plasma HS (*R* = 0.34, *P* = 0.08).

To understand this finding in more detail, we attempted to roughly compensate for the presence of heparin [which dose dependently blocks HPSE (18)] by using activated partial thromboplastin time (aPTT) as an adjustment. The aPTT was significantly prolonged in COVID-19 (**Table 1**) and correlated positively with HPSE activity (*R* = 0.58, *P* < 0.001), suggesting that the true HPSE activity in severely ill COVID-19 patients could be even higher than the values actually measured. Adjusting HPSE activity for aPTT (normalized HPSE/normalized aPTT quotient) indeed revealed a moderate association with the PBR (*R* = 0.44, *P* = 0.025).

**Figure 3:**
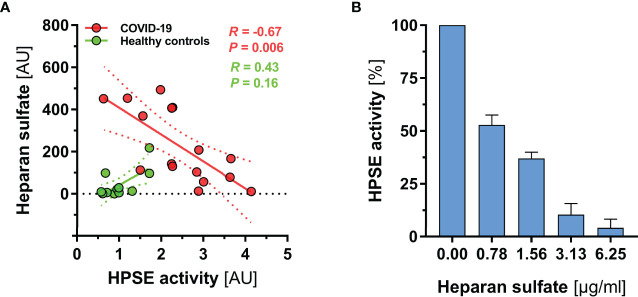
HS fragments in severe COVID-19 may partially block HPSE activity. **(A)** Scatter dot plot showing regression slopes of HPSE activity *vs*. HS plasma concentration in healthy subjects (n = 12) and COVID-19 patients (n = 16), respectively. **(B)** Bar charts showing percentage decrease of HPSE activity with increasing amounts of HS (isolated from bovine kidney) in three independent experiments. For this experiment, recombinant human HPSE was used in a concentration of 150 ng/ml. Data are presented as mean ± SEM.

Finally, we investigated the relationship between HPSE and HS in more detail. Interestingly, separate analysis of the two groups revealed contrasting regression slopes, suggesting that excessive generation of HS fragments in severe COVID-19 (which was not present in controls) may have partially blocked HPSE activity in COVID-19 patients (**Figure 3A**). Additional *in vitro* inhibition experiments with HS isolated from bovine kidney confirmed a dose dependent inhibition of HPSE at concentrations between 0 and 6.25 µg/ml; nearly full inhibition of heparanase was reached at a concentration of 6.25 µg/ml HS (**Figure 3B**).

## Discussion

Since the beginning of the COVID-19 pandemic, evidence has emerged that COVID-19 is a vascular rather than purely respiratory illness (1). Here we show that HPSE, the only enzyme in mammals that degrades HS chains from HS proteoglycans of the glycocalyx, plays a key role in mediating eGC damage in COVID-19.

HPSE is predominantly released by vascular endothelium, macrophages, and platelets. Its expression is up-regulated in endothelial cells by several factors, such as reactive oxygen species, inflammatory cytokines, high glucose, and advanced glycosylation products (22–25). Two previous COVID-19 studies measured HPSE antigen levels (26, 27) and two others determined enzymatic HPSE activity (18, 28). All but one study reported significantly elevated HPSE levels in COVID-19 patients. This finding therefore fits well with our previous data showing that sublingual eGC thickness (PBR) appears to be an appropriate risk marker for hospital mortality in COVID-19 (4). The *in vitro* experiments in our current study show that HPSE is a critical factor for eGC damage, as glycocalyx thinning correlated with HPSE activity and could be completely prevented by HPSE inhibition. The latter finding is even more surprising because the protective effect was achieved despite the presence of numerous cytokines and mediators in the patients’ sera (**Table 1**). This makes HPSE a promising target for intervention in COVID-19.

The possible involvement of HPSE in COVID-19 pathology was predicted already at the beginning of the pandemic long before initial studies on the subject (22, 29). It was speculated at the time that HPSE might contribute to the removal of HS chains from cell surfaces, facilitating virus release from host cells - a mechanism that has been well described for herpes simplex virus-1 (30, 31). Accordingly, enzymatic removal of HS from the eGC by HPSE is probably not the only underlying mechanism. For example, HPSE can induce matrix metalloproteases, which in turn cut transmembrane heparan sulfate proteoglycans such as syndecan-1 and thus further enhance eGC damage (31). Consistent with this assessment, our patients showed markedly elevated blood levels of hyaluronan and syndecan-1. The finding that HPSE activity in healthy individuals and COVID-19 patients was inversely correlated with HS concentration suggests that a minimum amount of HPSE is required before systemic eGC degradation begins. If so, HPSE would be more likely to be a late marker of disease severity. However, to disrupt the interaction of Sars-CoV-2 with the host cell, early blockade of HPSE could be beneficial.

In bacterial sepsis, the potential of HPSE and eGC as future pharmacological targets has already been highlighted (14, 15, 32). In detail, prevention of eGC damage by inhibition of HPSE significantly abolished vascular hyperpermeability and subsequent lung injury in murine endotoxemia (12). Using AFM we could show earlier, that unfractionated heparin (UFH), which also saturates HPSE, completely abolished the HPSE-induced decline of eGC thickness in freshly isolated rat aorta (20). Furthermore, the heparin fragment NAH prevented thinning of the eGC on human ECs induced by serum from sepsis patients *in vitro* (21). Based on these data, it would be very reasonable to assume that blocking HPSE may also be an effective approach to protecting eGC in COVID-19. Indeed, by western blotting of EC lysates, Potje et al. showed that low molecular weight heparin (LMWH) reduced the loss of glycocalyx components induced by plasma from COVID-19 patients (26). Our translational study comparing, for the first time, HPSE activity with the corresponding glycocalyx dimensions *in vivo* and *in vitro* in matched pairs strongly confirms and extends this exciting finding.

At the clinical level, the administration of heparin and/or LMWH has been shown to provide a significant survival benefit in both entities (33–35). Hence, apart from the anticoagulant effect of heparin the non-anticoagulant and, among these, especially anti-inflammatory properties should not be neglected and deserve closer attention (36). It is conceivable that this protective effect could be partly due to an off-target effect of heparin – i.e., the inhibition of HPSE. Further studies, ideally with accompanying HPSE measurement and sublingual video microscopy, are needed to confirm this assumption. However, the paradoxical decrease in HPSE activity in the COVID-19 patients with the highest HS concentrations certainly complicates any interpretation of HPSE activity in plasma samples. It is interesting to speculate that this could be a feedback loop whose aim is to keep HPSE activity within a certain range and to dampen its deleterious effects on the eGC. However, our data emphasize the importance of analyzing the HPSE in relation to the HS (and also to UFH/LMWH) in future studies. Of note, the investigation of heparins or heparin mimetics in COVID-19 appears particularly promising as these drugs hold the potential to counteract disease onset and progression in various ways. Not only the inhibition of endothelial HPSE, as presented in the current study, and HPSE of viral origin, as mentioned above, emerge as relevant target structures for heparins. Recent studies revealed HS/heparin-related binding sites on the SARS-CoV-2 spike protein and co-binding of HS and angiotensin-converting enzyme 2 seems to be necessary for docking to cellular surfaces. Heparins, on the other hand, were capable to potently block spike protein binding, probably due to blocking these specific binding sites (37). Consequently, not only heparins but also heparan sulfate mimetics – so far especially investigated in context of cancer therapy – come to interest as potential drugs in future COVID-19 treatment studies (38).

As our study is primarily hypothesis-generating, some limitations should be noted. First, the sample size of this proof-of-concept study was rather small. However, our dataset includes a wide range of glycocalyx and inflammatory markers, as well as a novel microscopy method to calculate the PBR *in vivo*. Although the PBR is not measured directly but estimated based on the radial displacement of red blood cells, this validated method is highly reliable (14, 16, 17, 39). Differences in PBR between patients and controls matched well with values obtained by Stahl et al. in another COVID-19 ICU cohort (27). Second, the use of aPTT as a measure of normalization of HPSE activity is certainly subject to its own variance. It would have been better to use either an inhibitor constant of heparin/LMWH or anti-Xa activity for correction to derive the active HPSE level. As we do not have citrate retain samples, a more accurate correction for heparin/LMWH effect was not possible in this data set. Unfortunately, the type, dose and application route of the heparins used were very heterogeneous, so that we were unable to convert these data into meaningful equivalent concentrations. Third, the number of serum samples used for the *in vitro* studies is limited because the AFM technique is sophisticated and time-consuming.

In conclusion, our translational study indicates that HPSE is a putative mediator of endothelial glycocalyx damage in COVID-19. Further studies are needed to clarify whether the benefit of heparin administration in COVID-19 is due to an eGC-protective off-target effect.

## Data Availability Statement

The raw data supporting the conclusions of this article will be made available by the authors, without undue reservation.

## Ethics Statement

The studies involving human participants were reviewed and approved by Ethics Committee of the General Medical Council Westfalen-Lippe and the WWU Münster, Germany. The patients/participants provided their written informed consent to participate in this study.

## Author Contributions

CD designed and conducted the *in vitro* experiments, analyzed the data, prepared the figures and contributed to the manuscript; AR and IO enrolled patients, performed sublingual video microscopy, performed ELISA assays and contributed to the manuscript; MR performed AFM experiments and revised the manuscript; BB, RS, and JV performed the HPSE activity and HS competition assays and contributed to the manuscript; AL, HP, and WL advised on the study design, discussed the findings and revised the manuscript; PK had the initial idea, supervised the study, recruited and coordinated participating centers, contributed to the analysis and figures, and drafted the manuscript. All authors contributed to the article and approved the submitted version.

## Funding

This work was supported by the German Research Foundation (rotational position of KFO 342 – ZA428/18-1 to CD and AR; KU 2873/3-1 to PK) and the fund Innovative Medical Research of the University of Münster Medical School (I-RO221907 to AR). The funding sources had no role in study design, data collection and analysis, the decision to publish, or preparation of the manuscript.

## Conflict of Interest

The authors declare that the research was conducted in the absence of any commercial or financial relationships that could be construed as a potential conflict of interest.

## Publisher’s Note

All claims expressed in this article are solely those of the authors and do not necessarily represent those of their affiliated organizations, or those of the publisher, the editors and the reviewers. Any product that may be evaluated in this article, or claim that may be made by its manufacturer, is not guaranteed or endorsed by the publisher.
